# Broadband and Efficient Envelope Amplifier for Envelope Elimination and Restoration/Envelope Tracking Higher-Efficiency Power Amplifiers

**DOI:** 10.3390/s22239173

**Published:** 2022-11-25

**Authors:** Oleg Varlamov, Dang Canh Nguyen, Andrei Grebennikov

**Affiliations:** 1 Department of Radio Equipment and Circuitry, Moscow Technical University of Communication and Informatics, 111024 Moscow, Russia; 2 Sumitomo Electric Europe Ltd., Elstree WD6 3SL, UK

**Keywords:** broadband DC amplifier, circuit optimization, envelope modulator, envelope tracking, envelope elimination and restoration, high-efficiency power amplifier, OFDM, probability distribution

## Abstract

Increasing the efficiency of transmitters, as the largest consumers of energy, is relevant for any wireless communication devices. For higher efficiency, a number of methods are used, including envelope tracking and envelope elimination and restoration. Increasing the bandwidth of used frequencies requires expanding envelope modulators bandwidth up to 250–500 MHz or more. The possibility of using amplifiers with input signal quantization (AISQ), as an alternative to the most common hybrid envelope tracking modulators, is considered. An approach has been developed for optimizing AISQ characteristics according to the criterion of minimum loss when amplifying modern telecommunication signals with Rayleigh envelope distribution. The optimal quantization levels are determined and the energy characteristics of AISQ are calculated. AISQ loss power is shown to decrease by 1.66 times with two-level quantization, by 2.4 times with three-level quantization, and by a factor of 3.0–3.7 for four–five quantization levels compared to a class B amplifier. With these parameters, AISQ becomes competitive with respect to hybrid envelope tracking modulators but does not have electromagnetic interference from the pulse width modulation (PWM) path.

## 1. Introduction

The trend of continuous growth in the number of communication systems subscribers [1] and television and radio broadcasting [2,3] systems is accompanied by an increase in data rates, and in some cases occurs with the frequency band usage expansion. Therefore, according to [4], at the end of 2021, there were around 8.2 billion mobile subscriptions, which will increase to around 9.1 billion by the end of 2027. The number of 5G mobile subscriptions is set to surpass 1 billion in 2022. By the end of 2027, 5G subscriptions are expected to reach 4.4 billion.

At the same time, there is a decrease in the specific power of single equipment with an increase in their total power. Thus, the total power consumption of the digital television broadcasting network transmitters of the Russian Federation DVB-T2 exceeded the total power of the disconnected high-power broadcasting network transmitters of the Russian Federation in the LF, MF, and HF bands [5]. With an increase in the cellular subscriber density, the radii of base station coverage areas decrease, and the power of the subscriber terminals decreases, but their number increases several times, which leads to a total increase in the radiated power. Under these conditions, the issues of building highly efficient power amplifiers, as the most energy-intensive parts of almost any radio equipment are of particular relevance.

All the main methods of modulated radio frequency (RF) signals with highly efficient amplification (outphasing modulation [6], envelope elimination and restoration (EER) [7]) were proposed in the 1930s–1950s. However, the constant increase in operating frequencies and transmission data rates with a corresponding expansion of the used frequency band, as well as the development of the element base, cause a continuously growing interest in these developments.

As the most common method of RF signals’ highly efficient amplification, it is considered to be an amplifier with envelope elimination and restoration (Kahn’s method) [7] and its “light” version, envelope tracking (ET). The issues of building its high-frequency (RF) path, including the use of classes D, E, and F switching power amplifiers, are widely covered in the literature, including by the authors [8]. This article discusses promising solutions for the construction of the modulation (envelope) path of EER/ET transmitters, namely, broadband direct current (DC) amplifiers (DCA).

Powerful broadband DC amplifiers are currently used in the modulation paths of both AM and SSB EER radio transmitters. The most common among such devices are pulse-width modulation (PWM) amplifiers [9]. A PWM amplifier generally consists of a pulse-width-modulated square wave generator, a switching amplifier, and an output low-pass filter (LPF) [9]. The use of the switching operating mode of the active elements provides high efficiency, little dependent on the amplitude of the amplified signal. At the same time, the presence of PWM conversion products in the output signal and a significant group delay of the amplified signal should be noted as disadvantages of such an amplifier. The need to ensure an acceptable level of PWM conversion products and the value of the group delay impose conflicting requirements on the frequency characteristics of the output LPF. Obviously, the noted factors did not prevent the use of PWM amplifiers in AM radio transmitters, where the amplified signal bandwidth did not exceed 10–12 kHz, and the LPF delay did not play a decisive role. In modern developments, the presence in the envelope path of a relatively narrow-band LPF is compensated by the introduction of a digital delay in the path of the high-frequency component formation. This factor no longer limits the amplification method under consideration application in the developed promising modifications of highly efficient EER switching amplifiers. The main limiting factor in the use of PWM amplifiers in amplifying the envelope of modern telecommunications and television and radio broadcasting signals is technological limitations on the element base speed, which, as a rule, decreases with an increase in the switched power. The use of multiphase PWM [9] and additional sigma-delta modulation [10] allows to slightly expand the boundaries of the PWM amplifier application areas. However, fundamental limits on switching speed remain.

The noted factor necessitates the search for other methods for constructing highly efficient broadband DCAs. This article provides a comparative analysis of various options for constructing a DCA, which differ from an amplifier with a PWM wider bandwidth and a significantly lower group delay of the amplified signal at high-energy performance. The option most suitable for practical implementation is selected, and an example of optimizing its parameters and calculating its energy characteristics is given.

The rest of this article is organized as follows. Section 2 provides some background information, including a discussion of amplifiers with switching and linear channels and amplifiers with input signal quantization (AISQ). In Section 3a choice of quantization characteristics optimization criteria is presented. An example of optimizing AISQ quantization characteristics on the signal $U(t)=U_{m}t$ (where U(t) is the instantaneous voltage value, $U_{m}$ is the maximum voltage value, and t is the current time) is considered, and AISQ energy parameters are analyzed. The optimization and analysis of the AISQ characteristics for modern telecommunication signals are carried out in Section 4. In Section 5, the main results are summarized, and the main conclusions are given.

## 2. Overview of High-Efficiency Low-Inertia DC Amplifiers Constructing Methods

In this section, we provide some necessary materials, including an overview of the methods for constructing high-efficiency, low-inertia DCAs.

### 2.1. Amplifiers with Switching and Linear Channel

**Figure 1:**
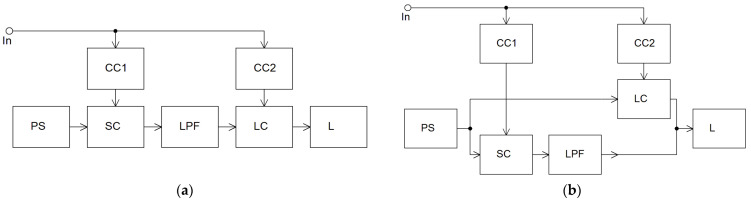
Structural diagrams of amplifiers with serial (**a**) and parallel (**b**) connections of linear and switching channels. CC—control circuit; PS—power supply; SC—switching channel; LPF—low pass filter; LC—linear channel; L—load.

One way to expand the bandwidth of high-efficiency DCAs is to use switching amplifiers with an additional linear channel. Amplifiers with switching and linear channels can be built both with the serial (Figure 1a) and parallel connections of channels to the load (Figure 1b) or with their combination [11].

In the case of the serial connection of channels, the entire amplifier can be considered as a linear amplifier with a switching channel for supply voltage regulation. With the help of a switching channel, at any level of the input signal, a relatively small voltage drop across the linear channel control element is maintained. Thereby, increased efficiency is ensured. This is somewhat similar to envelope tracking, only not by radio frequency but by the envelope. It should be noted that the overall efficiency of such an amplifier is defined as the multiplication of the linear and switching channels’ efficiency, so it is fundamentally less than in a class D amplifier with PWM. However, in this case, the requirements for the acceptable level of PWM products converted at the switching channel output can be reduced, since the linear channel performs its additional filtering. At the same time, the presence of a filter in the switching channel causes a relative delay of the signals in the linear and switching channels, and as the signal frequency increases, additional distortions arise due to this. To eliminate them, it is necessary to reduce the depth of the regulation of the linear amplifier supply voltage by the switching channel. However, in this case, the residual voltage on the linear channel transistors increases, and, accordingly, the overall efficiency decreases.

When the channels are connected in parallel, the main energy from the power source enters the load through the amplifier switching channel, and only a small part of it (to correct errors of the switching channel regulation) through the linear channel. This is the most common solution [12,13,14]. With this design of the amplifier, the overall efficiency is fundamentally higher than when the channels are connected in series; however, the level of distortion (with similar low-pass filter parameters) is higher. In addition, due to the presence of signal delay in the switching channel LPF, with an increase in the amplified signal frequency, it is necessary to increase the linear channel power, which also reduces the resulting efficiency.

Given the above, it can be noted that the combination of linear-switching channels, compared to amplifiers with PWM, has a lower efficiency. The use of a linear channel makes it possible to carry out additional filtering of the PWM conversion products, which leads to a reduction in the requirements for the LPF parameters and makes it possible to slightly reduce the group delay and expand the DCA bandwidth. At the same time, it is not possible to achieve large bandwidths (250–500 MHz) while maintaining a low level of distortion in such amplifiers. The noted factors limit the use of amplifiers with linear and switching channels as modulators of promising EER/ET amplifiers.

### 2.2. Amplifiers with Input Signal Quantization

When building high-efficiency broadband DCAs, along with PWM, the input signal quantization method can be used. Amplifiers with input signal quantization (AISQ) can be built in two ways: with quantization by the envelope of the amplified signal and with quantization by its instantaneous value [15].

AISQ with envelope quantization is described in detail in [15], where it is shown that the efficiency of such an amplifier is higher than that of a class “B” amplifier but lower than the efficiency of AISQ with instantaneous value quantization. In addition, AISQ with envelope quantization cannot in principle be freed from distortions of the “limitation” type of a part of the periods of the amplified signal [15], which makes it unsuitable for use as DCA modulators for EER/ET transmitters.

**Figure 2:**
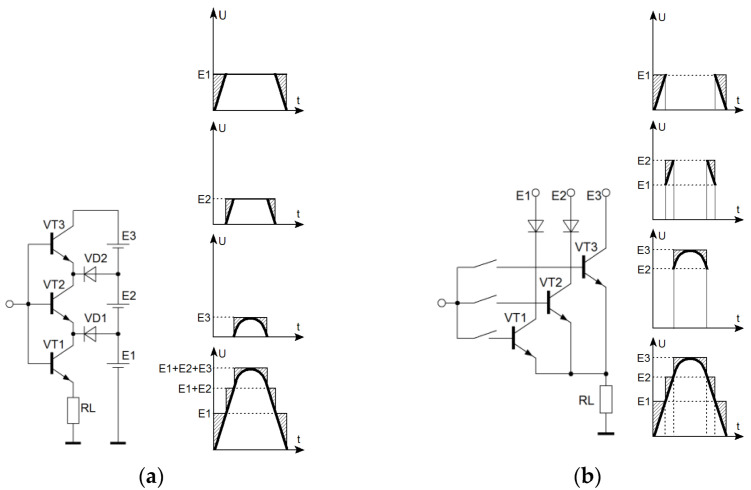
Diagrams of voltages of AISQ (shaded areas characterize losses): (**a**) with the serial connection of channels; (**b**) with parallel connection of channels. E1, E2, E3—quantization levels.

More promising for solving this problem is the use of AISQ with instantaneous value quantization, consisting of *n* amplifying cells with *n* power sources, each of which operates a part of the signal being amplified period. Representing the input signal as a sum of trapezoidal signals, and amplifying each component of this quantized signal with a class “B” amplifier, followed by summing the signals from the outputs of all amplifying cells, we obtain an amplified original signal in the load (Figure 2). It is quite obvious that with an increase in the number of quantization levels, the efficiency of the system as a whole increases since the ratio of the amplified pulse duration to its front duration increases, (Figure 2a) or there is an amplification of pulses with a flatter top (Figure 2b).

To reduce the commutation interference that occurs when switching amplifying channels, you can use a smooth redistribution of current in the load when switching from the previous channel to the next. Compared to PWM amplifiers, smooth channel switching allows, in addition to reducing the level of commutation interference, to reduce the requirements for the frequency properties of the amplifier active elements by about ten times. This makes it possible to achieve sufficiently large bandwidths (up to 500 MHz) while maintaining high efficiency. The issues of quantization level optimization and AISQ energy characteristics calculation depend on the amplified signal statistics. The optimization criteria are discussed in the next section of the article, which gives examples of determining the quantization levels and calculating the relative losses of AISQ.

## 3. AISQ Quantization Characteristics Optimization and Energy Parameters

### 3.1. Choice of Quantization Characteristics Optimization Criteria

When optimizing quantization levels, it must be remembered that AISQ, when used in the modulation path of an EER/ET power amplifier, is a DC amplifier. In general, a unipolar signal of any shape can be fed into its input, including a constant voltage in CW mode, a signal envelope with single-sideband modulation, or an OFDM signal envelope with various amplitude distributions in various applications. The noted factors determine the choice of criteria for quantization level optimization.

For example, AISQ can be used in the EER amplifier low-frequency path, which should amplify the signal with a constant carrier for a sufficiently long time—for example, in the telegraph (CW) mode of operation. In this case, it is necessary to optimize the quantization levels according to the criterion of minimizing the highest value of the power loss of the amplifier, depending on the signal level in the static mode.

If the signal amplification mode with single-sideband modulation is mainly used, then it is advisable to optimize the quantization levels by minimizing the average loss power of the entire AISQ as a whole. In this case, the envelope of a two-tone equal-amplitude signal should be used as the input signal model: $U(t)=U_{m}|\cos\omega t|$, which is the standard test signal.

When using AISQ as a modulation path of an EER/ET amplifier of modern telecommunication signals of communication and broadcasting with OFDM modulation, it is necessary to take into account the amplitude distribution of a particular application.

The use of the above criteria when calculating quantization levels will allow, in addition to increasing the efficiency, to minimize the volume and mass of the AISQ cooling system, improve the thermal conditions of its output stages, and thereby increase the reliability of the amplifier as a whole.

Taking into account the wide variety of different options for using the AISQ and the corresponding procedures for optimizing the quantization thresholds, as an example, we will optimize the quantization levels according to the criterion of minimizing the average loss power when amplifying the signal of the form: $U(t)=U_{m}t$.

### 3.2. An Example of Optimizing AISQ Quantization Characteristics

**Figure 3:**
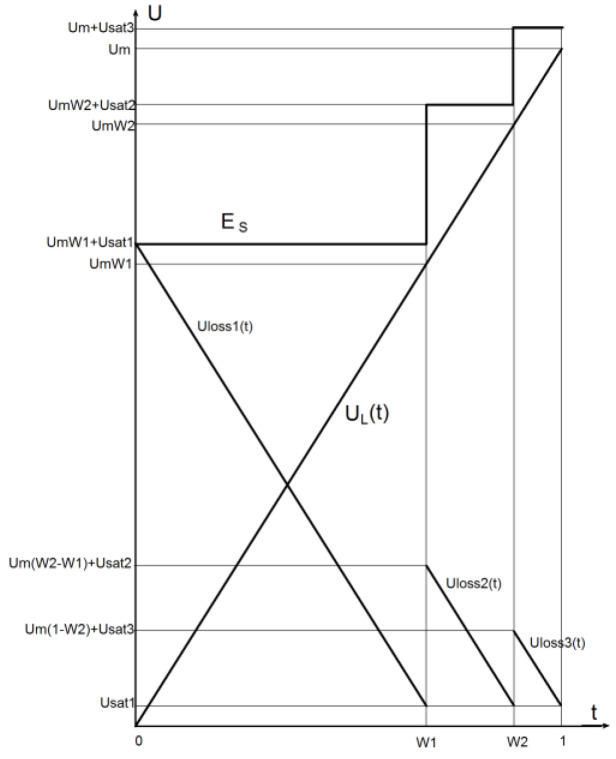
Plots of 3-level AISQ voltages with amplification of the $U(t)=U_{m}t$ signal form. $U_{lossi}(t)$ indicates losses in the i-th stage.

To optimize AISQ quantization characteristics according to the criterion of minimizing the average power loss when amplifying the signal of the form $U(t)=U_{m}t$, in accordance with Figure 3, the authors can write down the laws of change in the normalized voltage at the load $U_{L}(t)$ and transistors of various stages:

(1) $$U_{L}(t)=U_{m}t,$$

(2) $$\begin{array}{l} U_{T1}(t)=U_{m}W_{1}+U_{Sat1}-U_{L}(t),\;\;\;\;\;\;\;\;\;\;\;\;\;\;\;U_{L}\in \left[0;\;\;\;U_{m}W_{1}\right]\\ \cdots \cdots \cdots \\ U_{Ti}(t)=U_{m}W_{i}+U_{Sati}-U_{L}(t),\;\;\;\;\;\;\;\;\;\;\;\;\;\;\;\;\;U_{L}\in \left[U_{m}W_{i-1};\;\;\;U_{m}W_{1}\right],\\ \cdots \cdots \cdots \\ U_{Tn}(t)=U_{m}+U_{Satn}-U_{L}(t),\;\;\;\;\;\;\;\;\;\;\;\;\;\;\;\;\;U_{L}\in \left[U_{m}W_{n-1};\;\;\;1\right] \end{array}$$

where *t* is the relative time equal to the ratio of the current time to the signal period;

$U_{Ti}(t)$—collector-emitter voltage of the *i*-th stage transistor $\left(U_{Ti}(t)=E_{Si}-U_{L}(t)\right)$;

$E_{Si}$—supply voltage of the *i*-th stage;

$U_{m}$—maximum voltage on the load;

$W_{i}$—*i*-th relative quantization level $\left(W_{i}=(E_{Si}-U_{Sati})/U_{m}\right)$;

$U_{Sati}$—saturation voltage on the *i*-th stage transistor $\left(U_{Sati}=E_{Si}-U_{m}W_{i}\right)$;

*n* is the number of quantization levels.

Based on (2), it is possible to determine the laws of change in the power losses that are allocated in the output stages of the AISQ:

(3) $$\begin{array}{l} P_{LOSS1}(W_{1})=\frac{1}{T}\int_{0}^{T}U_{T1}(t)i_{T1}(t)dt=\int_{0}^{t_{1}}U_{T1}(t)\frac{U_{L}(t)}{R_{L}}dt=\frac{1}{R_{L}}\int_{0}^{t_{1}}(U_{m}W_{1}+U_{Sat1}-U_{m}t)U_{m}tdt=;\\ \frac{U_{m}^{2}}{R_{L}}\int_{0}^{t_{1}}\left(W_{1}t+y_{1}t-t^{2}\right)dt=\frac{U_{m}^{2}}{R_{L}}(\frac{W_{1}^{3}}{6}+y_{1}\frac{W_{1}^{2}}{2});\\ \;P_{LOSS2}(W_{1},W_{2})=\frac{1}{T}\int_{0}^{T}U_{T2}(t)i_{T2}(t)dt=\frac{1}{R_{L}}\int_{t_{1}}^{t_{2}}(U_{m}W_{2}+U_{Sat2}-U_{m}t)U_{m}tdt=;\\ \;\frac{U_{m}^{2}}{R_{L}}(\frac{W_{2}^{3}}{6}+\frac{W_{1}^{3}}{3}+y_{2}\frac{W_{2}^{2}}{2}-\frac{W_{1}^{2}W_{2}}{2}-y_{2}\frac{W_{1}^{2}}{2});\\ \;\;\cdots \cdots \cdots \\ \;\;P_{LOSSi}(W_{i-1},W_{i})=\frac{U_{m}^{2}}{R_{L}}(\frac{W_{i}^{3}}{6}+\frac{W_{i-1}^{3}}{3}+y_{i}\frac{W_{i}^{2}}{2}-\frac{W_{i-1}^{2}W_{i}}{2}-y_{i}\frac{W_{i-1}^{2}}{2}), \end{array}$$

where $y_{i}$—*i*-th normalized saturation voltage $\left(y_{i}=U_{Sati}/U_{m}\right)$;

$P_{LOSSi}(W_{i-1},W_{i})$—loss power released in the *i*-th stage of the amplifier;

$W_{i}$—phase of the *i*-th level of quantization, and $W_{0}=0$, $W_{n}=1$.

The total power loss in all stages of the amplifier:

(4) $$P_{LOSS\Sigma }(W_{1},\ldots W_{n-1})=\frac{U_{m}^{2}}{2R_{L}}\left\{y_{n}+\frac{1}{3}+\sum_{i=1}^{n-1}\left[W_{i}^{2}(y_{i}-y_{i+1})+W_{i}^{3}-W_{i+1}W_{i}^{2}\right]\right\}.$$

To find the optimal quantization levels at which the average power loss of the AISQ is minimal, it is necessary to find the partial derivatives of $P_{LOSS\Sigma }(W_{1},\ldots W_{n-1})$ and equate them to zero:

(5) $$\left\{ \begin{array}{l} \frac{\partial P_{LOSS\Sigma }(W_{1},\ldots W_{n-1})}{\partial W_{1}}=0\\ \cdots \cdots \cdots \\ \frac{\partial P_{LOSS\Sigma }(W_{1},\ldots W_{n-1})}{\partial W_{n-1}}=0 \end{array}\right..$$

Substituting (4) into (5), we find:

(6) $$\left\{ \begin{array}{l} (y_{1}-y_{2})W_{1}+1.5W_{1}^{2}-W_{1}W_{2}=0\\ \cdots \cdots \cdots \\ (y_{i}-y_{i+1})W_{i}+1.5W_{i}^{2}-W_{i}W_{i+1}-\frac{W_{i-1}}{2}=0 \end{array}\right..$$

The solution of system (6) for large *n* is not complicated in a general form but is rather cumbersome. As an example, for *n* = 3, it can be shown that

(7) $$\begin{array}{l} W_{2}=(y_{1}-y_{2})+1.5W_{1}\\ W_{3}=(y_{2}-y_{3})+1.5(y_{1}-y_{2})+2.25W_{1}-\frac{W_{1}^{2}}{2(y_{1}-y_{2})+3W_{1}} \end{array}.$$

At the same time, assuming for simplification, $U_{Sat1}=\cdots =U_{Satn}$, which is easily achieved with a rational choice of the element base of individual cells and will not lead to a noticeable error, due to $E_{S}\gg U_{Sat}$, it is possible to determine from (6) the location of the optimal quantization levels:

(8) $$\begin{array}{l} W_{2}=1.5W_{1}\\ W_{3}=1.92W_{1}\\ W_{4}=2.29W_{1}\\ W_{5}=2.63W_{1}\\ W_{6}=2.95W_{1} \end{array}$$

From (8), it follows that the location of the optimal quantization levels does not depend on the saturation voltages when they are equal in the individual amplifying stages. The results of calculating the optimal quantization levels for various *n* are presented in Table 1.

### 3.3. AISQ Energy Parameters Analysis

To determine AISQ energy parameters, we use the expression (4), which describes the total power loss in all stages of the amplifier. Transforming it, taking into account these assumptions, we obtain:

(9) $$P_{LOSS\Sigma }(W_{1},\ldots W_{n-1})=\frac{U_{m}^{2}}{2R_{L}}\left\{y_{n}+\frac{1}{3}+\sum_{i=1}^{n-1}\left[W_{i}^{3}-W_{i}^{2}W_{i+1}\right]\right\}.$$

**Figure 4:**
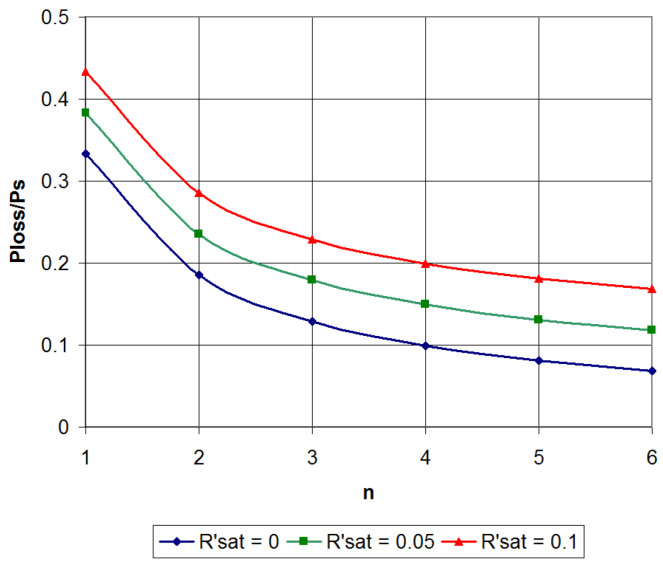
AISQ average power loss.

Substituting the values of the found optimal quantization levels into (9), after normalization, we obtain the relative average loss power for a different number of quantization levels (Figure 4). As can be seen from the figure, in comparison with a class B amplifier (*n* = 1), even with three-level quantization, the power loss in the AISQ decreases by 2–2.5 times (depending on the transistor’s saturation voltage). A further increase in *n* leads to a less sharp decrease in the relative power loss, which, with the obvious complication of the circuitry implementation of such AISQ, makes the use of three–four-level quantization the most expedient.

The considered example concerned a hypothetical case for amplifying a simple signal of the form $U(t)=U_{m}t$, for which analytical calculations can be carried out. The approach to calculating the AISQ characteristics will be discussed in the next section.

## 4. Discussion: AISQ for Modern Telecommunication Signals

Modern telecommunication signals, in particular the 5G NR standard, continuously increase their maximum channel bandwidth for intra-band contiguous carrier aggregation to improve their data rate. Briefly, 3GPP TS 38.101 (Release 16) introduces maximum aggregated BW cases of 200 MHz (CA_n77C, CA_n78C, CA_n79C), 300 MHz (CA_n77D, CA_n78D, CA_n79D) and even 320 MHz (CA_n46E) [16]. In response to these requirements, various solutions are being developed for building a broadband Envelope-Tracking Supply Modulator [17,18].

OFDM is by far the most common waveform, being used in both the cellular and 802.11 standards, and exhibits a Rayleigh amplitude distribution.

A structure similar to AISQ, considered in [19], does not contain optimization procedures for choosing quantization thresholds for a signal with Rayleigh amplitude distribution. In [19], they are chosen empirically around the “center of normalized mean voltage” based on assumptions.

Loss analysis for a signal with a Rayleigh amplitude distribution for an envelope amplifier with a combination of linear and switching channels is given in [20].

**Figure 5:**
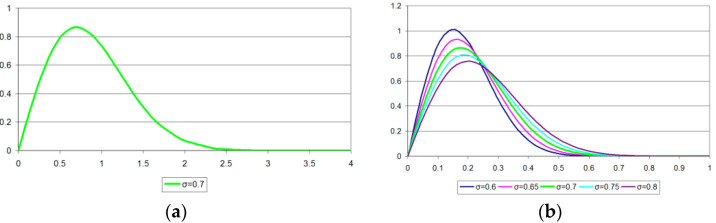
Envelope signal Rayleigh amplitude distribution: (**a**) with σ=0.7; (**b**) normalized to the amplitude interval [0;1] for a typical σ∈[0.6;0.8].

For an envelope signal with Rayleigh amplitude distribution $f(x,\sigma )=\frac{x}{\sigma^{2}}e^{-\frac{x^{2}}{2\sigma^{2}}}$ (Figure 5a) normalized to the amplitude interval [0;1] with negligible compression for a typical σ∈[0.6;0.8] as: $f^{\prime }(x,\sigma )=\frac{4x}{\sigma^{2}}e^{-\frac{16x^{2}}{2\sigma^{2}}}$ (Figure 5b), let us write down the expressions for the losses. To simplify the calculations, we take $U_{Sati}=0$. In this case, $W_{i}=u_{i}$.

For AISQ with two quantization levels, the losses in the first and second stages are:

(10) $$\begin{array}{l} P_{LOSS1}(u_{1})=\frac{1}{U}\int_{0}^{U}U_{T1}(x)i_{T1}(x)dx=\int_{0}^{u_{1}}U_{T1}(x)\frac{U_{L}(x)}{R_{L}}dx=\frac{1}{R_{L}}\int_{0}^{u_{1}}(u_{1}-x)x\frac{4x}{\sigma^{2}}e^{-\frac{16x^{2}}{2\sigma^{2}}}dx;\\ P_{LOSS2}(u_{1})=\frac{1}{U}\int_{0}^{U}U_{T2}(x)i_{T2}(x)dx=\int_{u_{1}}^{1}U_{T2}(x)\frac{U_{L}(x)}{R_{L}}dx=\frac{1}{R_{L}}\int_{u_{1}}^{1}(1-x)x\frac{4x}{\sigma^{2}}e^{-\frac{16x^{2}}{2\sigma^{2}}}dx. \end{array}$$

Integrating, we receive:

(11) $$\begin{array}{c} P_{LOSS1}(u_{1})=\frac{\sqrt{\pi }\sigma u_{1}erf\left(\frac{2^{3/2}u_{1}}{\sigma }\right)}{2^{9/2}}+\frac{\sigma^{2}e^{-\frac{8u_{1}^{2}}{\sigma^{2}}}-\sigma^{2}}{32};\\ P_{LOSS2}(u_{1})=\frac{e^{-\frac{8}{\sigma^{2}}}\left(2\sqrt{\pi }\sigma^{3}e^{\frac{8}{\sigma^{2}}}erf\left(\frac{2^{3/2}}{\sigma }\right)+\sqrt{2}\sigma^{4}\right)-e^{-\frac{8u_{1}^{2}}{\sigma^{2}}}\left(2\sqrt{\pi }\sigma^{3}e^{\frac{8u_{1}^{2}}{\sigma^{2}}}erf\left(\frac{2^{3/2}u_{1}}{\sigma }\right)+2^{7/2}\sigma^{2}u_{1}^{2}-2^{7/2}\sigma^{2}u_{1}^{}+\sqrt{2}\sigma^{4}\right)}{2^{11/2}\sigma^{2}}. \end{array}$$

Total losses:

(12) $$P_{LOSS\Sigma }(u_{1})=P_{LOSS1}(u_{1})+P_{LOSS2}(u_{1}).$$

Find the derivative of the total losses with respect to *u*_1_ (*u*_1_ = *x*)

(13) $$\frac{dP_{LOSS\Sigma }}{du_{1}}=\frac{e^{-\frac{8u_{1}^{2}}{\sigma^{2}}}\left(8\sqrt{\pi }\sigma^{3}e^{\frac{8u_{1}^{2}}{\sigma^{2}}}erf\left(\frac{2^{3/2}u_{1}}{\sigma }\right)+2^{19/2}u_{1}^{3}-2^{19/2}u_{1}^{2}-2^{11/2}\sigma^{2}u_{1}\right)}{2^{15/2}\sigma^{2}}.$$

Equating to zero and finding the roots of the equation, we find the optimal threshold values $u_{1}$ for a number of values σ in the interval σ∈[0.6;0.8] (Table 2).

Similarly, for an AISQ with three quantization levels, the stages of loss are:

(14) $$\begin{array}{l} P_{LOSS1}(u_{1})=\frac{1}{U}\int_{0}^{U}U_{T1}(x)i_{T1}(x)dx=\int_{0}^{u_{1}}U_{T1}(x)\frac{U_{L}(x)}{R_{L}}dx=\frac{1}{R_{L}}\int_{0}^{u_{1}}(u_{1}-x)x\frac{4x}{\sigma^{2}}e^{-\frac{16x^{2}}{2\sigma^{2}}}dx;\\ P_{LOSS2}(u_{1},u_{2})=\frac{1}{U}\int_{0}^{U}U_{T2}(x)i_{T2}(x)dx=\int_{u_{1}}^{u_{2}}U_{T2}(x)\frac{U_{L}(x)}{R_{L}}dx=\frac{1}{R_{L}}\int_{u_{1}}^{u_{2}}(u_{2}-x)x\frac{4x}{\sigma^{2}}e^{-\frac{16x^{2}}{2\sigma^{2}}}dx;\\ P_{LOSS3}(u_{2})=\frac{1}{U}\int_{0}^{U}U_{T2}(x)i_{T2}(x)dx=\int_{u2}^{1}U_{T2}(x)\frac{U_{L}(x)}{R_{L}}dx=\frac{1}{R_{L}}\int_{u_{2}}^{1}(1-x)x\frac{4x}{\sigma^{2}}e^{-\frac{16x^{2}}{2\sigma^{2}}}dx. \end{array}$$

Integrating, we find:

(15) $$\begin{array}{c} P_{LOSS1}(u_{1})=\frac{\sqrt{\pi }\sigma u_{1}erf\left(\frac{2^{3/2}u_{1}}{\sigma }\right)}{2^{9/2}}+\frac{\sigma^{2}e^{-\frac{8u_{1}^{2}}{\sigma^{2}}}-\sigma^{2}}{32};\\ P_{LOSS2}(u_{1},u_{2})=\frac{e^{-\frac{8u_{2}^{2}}{\sigma^{2}}}\left(2\sqrt{\pi }\sigma^{3}u_{2}e^{\frac{8u_{2}^{2}}{\sigma^{2}}}erf\left(\frac{2^{3/2}u_{2}}{\sigma }\right)+\sqrt{2}\sigma^{4}\right)-e^{-\frac{8u_{1}^{2}}{\sigma^{2}}}\left(2\sqrt{\pi }\sigma^{3}e^{\frac{8u_{1}^{2}}{\sigma^{2}}}erf\left(\frac{2^{3/2}u_{1}}{\sigma }\right)u_{2}-2^{7/2}\sigma^{2}u_{1}^{}u_{2}+2^{7/2}\sigma^{2}u_{1}^{2}+\sqrt{2}\sigma^{4}\right)}{2^{11/2}\sigma^{2}};\\ P_{LOSS3}(u_{2})=\frac{e^{-\frac{8}{\sigma^{2}}}\left(2\sqrt{\pi }\sigma^{3}e^{\frac{8}{\sigma^{2}}}erf\left(\frac{2^{3/2}}{\sigma }\right)+\sqrt{2}\sigma^{4}\right)-e^{-\frac{8u_{2}^{2}}{\sigma^{2}}}\left(2\sqrt{\pi }\sigma^{3}e^{\frac{8u_{2}^{2}}{\sigma^{2}}}erf\left(\frac{2^{3/2}u_{2}}{\sigma }\right)+2^{7/2}\sigma^{2}u_{2}^{2}-2^{7/2}\sigma^{2}u_{2}^{}+\sqrt{2}\sigma^{4}\right)}{2^{11/2}\sigma^{2}}. \end{array}$$

Total losses:

(16) $$P_{LOSS\Sigma }(u_{1},u_{2})=P_{LOSS1}(u_{1})+P_{LOSS2}(u_{1},u_{2})+P_{LOSS3}(u_{2}).$$

Partial derivatives of total losses with respect to *u*_1_ and *u*_2_:

(17) $$\begin{array}{c} \frac{dP_{LOSS\Sigma }}{du_{1}}=\frac{e^{-\frac{8u_{1}^{2}}{\sigma^{2}}}\left(\sqrt{2}\sqrt{\pi }\sigma^{3}e^{\frac{8u_{1}^{2}}{\sigma^{2}}}erf\left(\frac{2^{3/2}u_{1}}{\sigma }\right)+128u_{1}^{3}-128u_{2}u_{1}^{2}-8\sigma^{2}u_{1}\right)}{32\sigma^{2}};\\ \frac{dP_{LOSS\Sigma }}{du_{2}}=\frac{e^{-\frac{8(u_{1}^{2}+u_{2}^{2})}{\sigma^{2}}}\left(\sqrt{2}\sqrt{\pi }\sigma^{3}e^{\frac{8(u_{1}^{2}+u_{2}^{2})}{\sigma^{2}}}\left(erf\left(\frac{2^{3/2}u_{2}}{\sigma }\right)-erf\left(\frac{2^{3/2}u_{1}}{\sigma }\right)\right)+8\sigma^{2}u_{1}e^{\frac{8u_{2}^{2}}{\sigma^{2}}}+128e^{\frac{8u_{1}^{2}}{\sigma^{2}}}u_{2}^{3}-128e^{\frac{8u_{1}^{2}}{\sigma^{2}}}u_{2}^{2}-8\sigma^{2}u_{2}e^{\frac{8u_{1}^{2}}{\sigma^{2}}}\right)}{32\sigma^{2}}. \end{array}$$

Equation (17) can be found numerically for specific *σ* values. As an example, we can show quantization thresholds optimal values for *σ* = 0.7: $u_{1}=0.292$; $u_{2}=0.47$.

**Figure 6:**
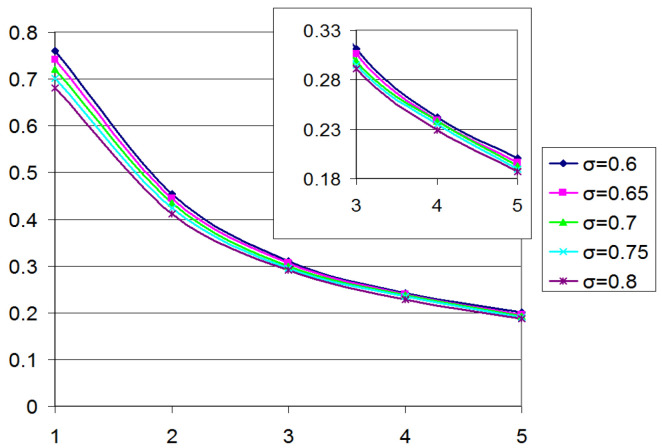
AISQ average power loss for envelope signal with Rayleigh amplitude distribution for a typical σ∈[0.6;0.8].

Using a similar approach, you can find the optimal quantization levels values for AISQ with four, five, etc. quantization levels (Table 3). Knowing the values of the optimal quantization levels, it is possible to determine the power loss using expressions similar to (12) and (16). Normalizing the power loss to the useful signal average power as $P_{LOSS\_REL}=P_{LOSS\Sigma }/(P_{SIG}+P_{LOSS\Sigma })$, we obtain the relative loss values. The calculation results of the relative losses during the amplification signal with the Rayleigh envelope distribution for σ∈[0.6;0.8] in AISQ with the number of quantization levels *n* = 1…5 are shown in Figure 6. Recall that *n* = 1 corresponds to a class B amplifier.

As can be seen from Figure 6, when amplifying a signal with Rayleigh envelope distribution, compared to a class B amplifier (*n* = 1), AISQ loss power decreases by 1.66 times with two-level quantization, by 2.4 times with three-level quantization, and by a factor of 3.0–3.7 for four–five quantization levels, weakly depending on the *σ*. A further increase in the quantization levels number is impractical due to the increase in the amplifier complexity.

## 5. Conclusions

Amplifiers with input signal quantization can be used as powerful broadband DC amplifiers in the modulation (envelope) path for promising EER/ET transmitters. To obtain the high-energy characteristics of AISQ, it is advisable to use a circuit with a parallel connection of its output channels, the switching of amplifying cells along the input circuits, and reducing the output transistor saturation voltage. AISQ, due to the absence of reactive elements in it, can be formed in an integrated design.

An approach has been developed for optimizing AISQ characteristics according to the criterion of minimum loss when amplifying modern telecommunication signals with Rayleigh envelope distribution. 

The optimal quantization levels are determined, and the energy characteristics of AISQ are calculated. AISQ power loss is shown to decrease by 1.66 times with two-level quantization, by 2.4 times with three-level quantization, and by a factor of 3.0–3.7 for four–five quantization levels compared to a class B amplifier. The obtained theoretical results show that with four-level optimized quantization for a signal with a peak-to-average power ratio (PAPR) of 12 dB, an efficiency of 76% is achieved. This is better than known experimental results [19] with empirically chosen quantization levels, where a similar efficiency was obtained for a signal with PARP 8.5 dB.

With these parameters, AISQ becomes competitive with respect to hybrid envelope tracking modulators but does not have electromagnetic interference from the PWM path.

## Figures and Tables

**Table 1:** Optimum quantization levels for the $U_{L}(t)=U_{m}t$ signal.

Quantization Level Number	Number of Quantization Levels				
	2	3	4	5	6
1	0.667	0.513	0.437	0.380	0.339
2	1.0	0.783	0.655	0.570	0.509
3	-	1.0	0.838	0.729	0.650
4	-	-	1.0	0.870	0.776
5	-	-	-	1.0	0.892
6	-	-	-	-	1.0

**Table 2:** Optimum quantization levels for various *σ*.

Quantization Level Number	*σ*				
	0.6	0.65	0.7	0.75	0.8
1	0.376	0.3976	0.418	0.4373	0.4557
2	1	1	1	1	1

**Table 3:** Optimum quantization levels for various *σ*.

Number of Quantization Levels	Quantization Level Number	*σ*				
		0.6	0.65	0.7	0.75	0.8
1	(Class B)	1	1	1	1	1
2	1	0.376	0.3976	0.418	0.4373	0.4557
	2	1	1	1	1	1
3	1	0.26	0.28	0.292	0.3	0.32
	2	0.436	0.456	0.47	0.485	0.505
	3	1	1	1	1	1
4	1	0.216	0.216	0.25	0.281	0.289
	2	0.313	0.33	0.344	0.38	0.396
	3	0.478	0.483	0.514	0.538	0.581
	4	1	1	1	1	1
5	1	0.196	0.206	0.216	0.24	0.261
	2	0.3	0.313	0.319	0.333	0.358
	3	0.401	0.411	0.414	0.416	0.453
	4	0.525	0.531	0.534	0.577	0.608
	5	1	1	1	1	1

## Data Availability

Not applicable.
